# Supplementary material for: Circulating trophoblast cell clusters for early detection of placenta accreta spectrum disorders
Source: Nat Commun. 2021 Aug 3;12:4408. doi: 10.1038/s41467-021-24627-2 (PMC8333096; doi:10.1038/s41467-021-24627-2)
Supplement: Supplementary file 1 — Supplementary Information [file 41467_2021_24627_MOESM1_ESM.pdf]

Supplementary Information

Circulating trophoblast cell clusters for early detection of placenta accreta spectrum disorders

Yalda Afshar†, Jiantong Dong†, Pan Zhao, Lei Li, Shan Wang, Ryan Y. Zhang, Ceng Zhang, Ophelia Yin, Christina S. Han, Brett D. Einerson, Tania L. Gonzalez, Huirong Zhang, Anqi Zhou, Zhuo Yang, Shih-Jie Chou, Na Sun, Ju Cheng, Henan Zhu, Jing Wang, Tiffany X. Zhang, Yi-Te Lee, Jasmine J. Wang, Pai-Chi Teng, Peng Yang, Dongping Qi, Meiping Zhao, Myung-Shin Sim, Ruilian Zhe, Jeffrey D. Goldstein, John Williams III, Xietong Wang, Qingying Zhang, Lawrence D. Platt, Chang Zou*, Margareta D. Pisarska*, Hsian-Rong Tseng*, Yazhen Zhu*

† These authors contributed equally: Yalda Afshar, Jiantong Dong.
*The authors jointly supervised this work: Hsian-Rong Tseng, Yazhen Zhu, Margareta D. Pisarska, Chang Zou. E-mail: hrtseng@mednet.ucla.edu (H.-R.T.); yazhenzhu@mednet.ucla.edu (Y.Z.); margareta.pisarska@cshs.org (M.P.); zou.chang@szhospital.com (C.Z.).

This PDF file includes:

Supplementary Figures 1 to 9

Supplementary Tables 1 to 5

Supplementary Note 1

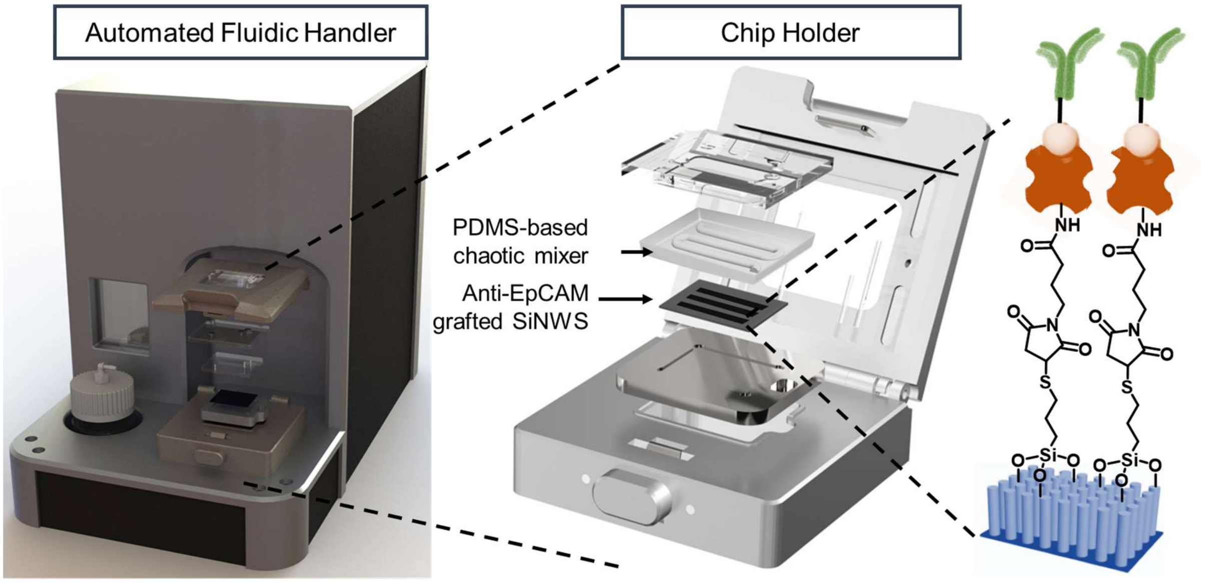

Supplementary Fig. 1. A photograph and a schematic diagram showing the entire NanoVelcro device.

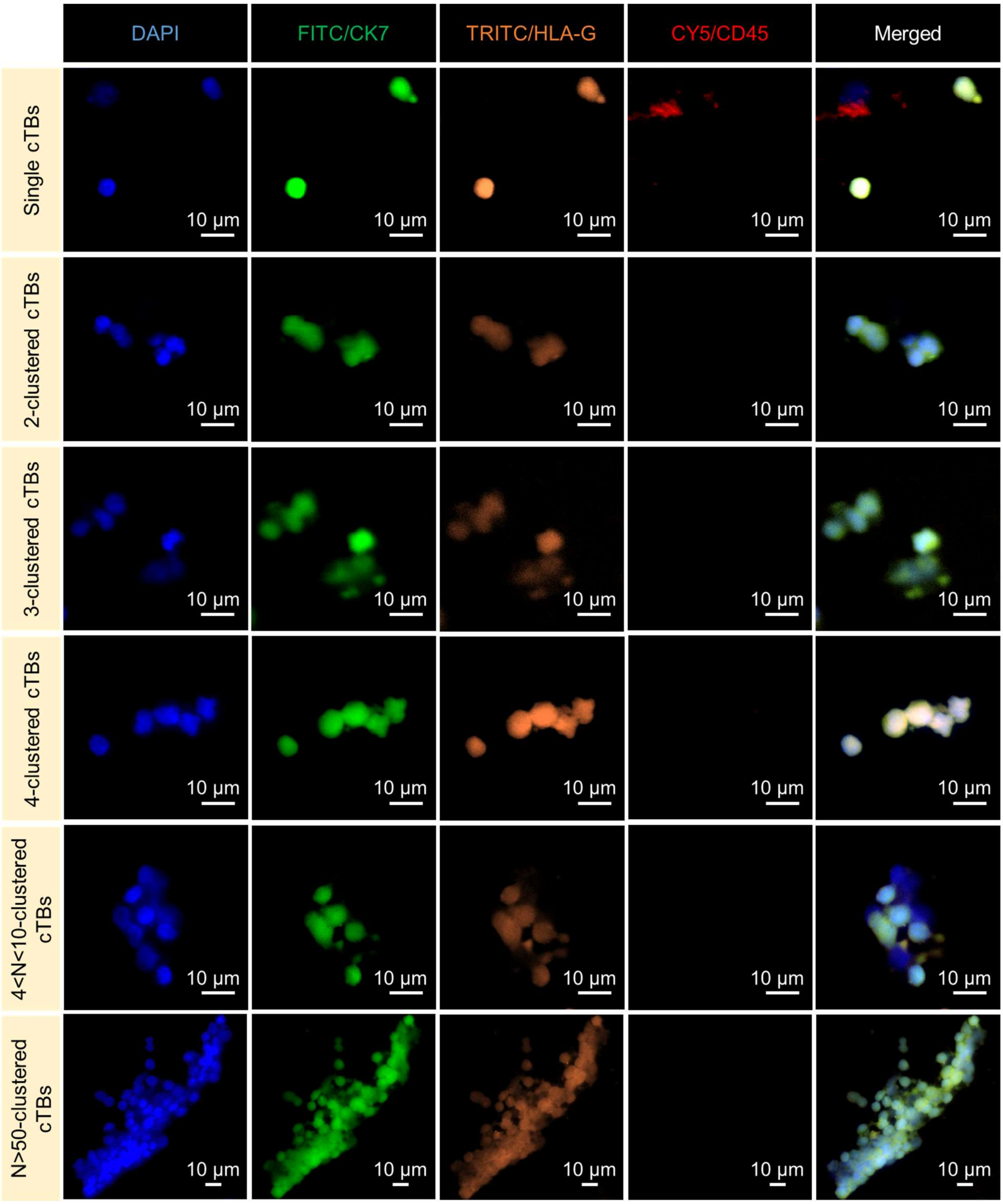

Supplementary Fig. 2. Representative images of single cTBs and cTB-clusters of varying cell numbers captured by NanoVelcro Chips. Blue: DAPI stained nuclei; green: FITC stained CK7; orange: TRITC stained HLA-G; red: CY5 stained CD45. Scale bar, 10 μm. Data are representatives of six independent assays.

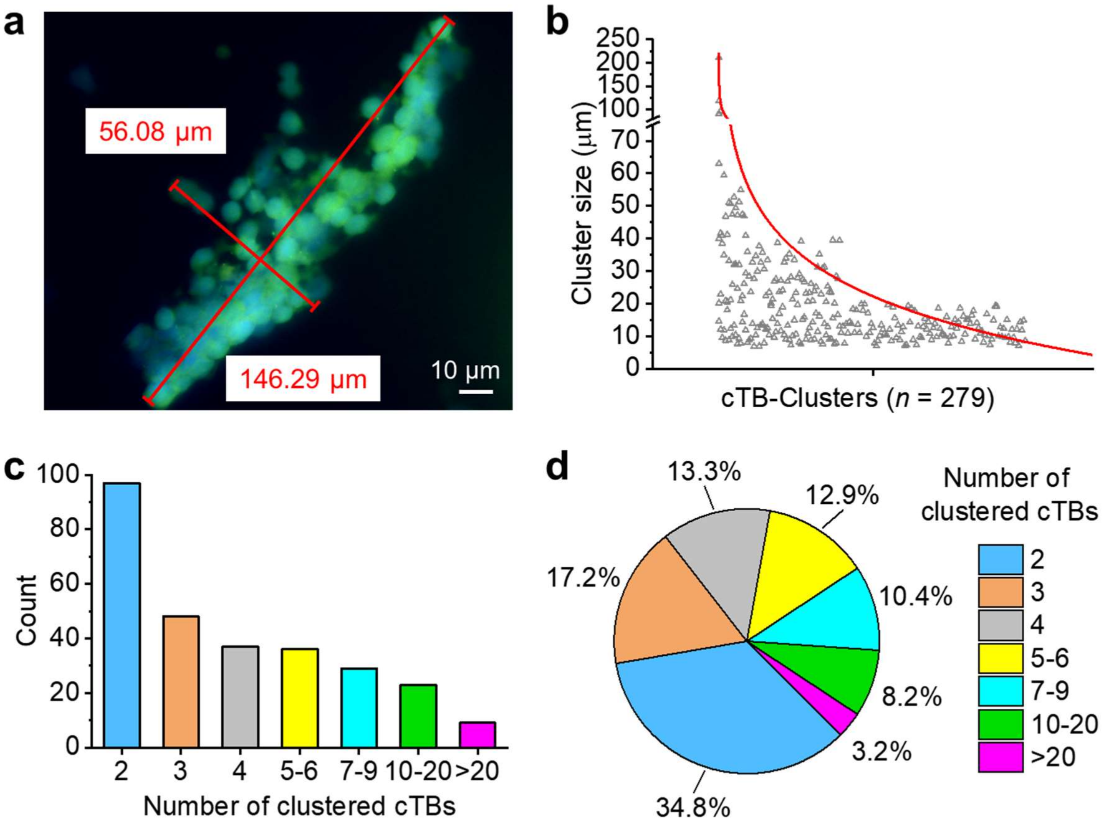

Supplementary Fig. 3. Size characterization of cTB-clusters captured by NanoVelcro Chips. **a** The size of a cTB-clusters was measured along the longest axis and width perpendicular to that axis across the cTB-clusters, defined as $\sqrt{(longest\ axis) \times (perpendicular\ width)}$. Scale bar, 10 µm. Data are representatives of three independent assays. **b** Plots showing the cluster size range of cTB-clusters (n = 279). **c** Bars and **d** pie charts showing the distribution and proportion of cTB-clusters (n = 279) with varying numbers of cells, respectively. Source data are provided in the Source Data file.

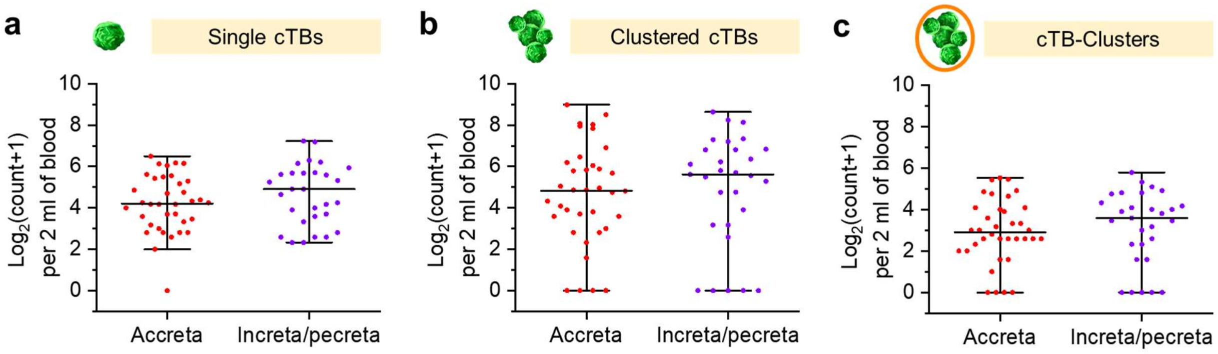

Supplementary Fig. 4. Counts of single and clustered cTBs as well as cTB-clusters based on stratification of placenta accreta spectrum (PAS) by accreta (*n* = 36), increta and percreta (*n* = 29): a single cTBs, **b** clustered cTBs, **c** cTB-clusters. Counts were log2-transformed. The two shorter horizontal lines denote the 25–75% interquartile ranges (IQR) and the longer horizontal lines in between denote the median. Data are expressed as Mean ± SE for **a** single cTBs: accreta (4.2 ± 0.2), increta and pecreta (4.6 ± 0.3); **b** clustered cTBs: accreta (4.5 ± 0.4), increta and pecreta (4.9 ± 0.5); and **c** cTB-clusters: accreta (2.9 ± 0.3), increta and pecreta (3.1 ± 0.3). Source data are provided in the Source Data file.

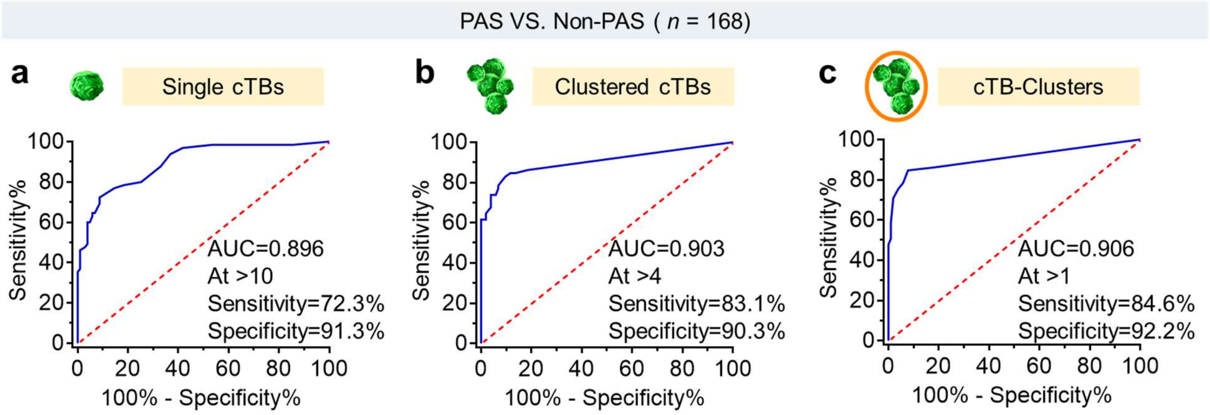

Supplementary Fig. 5. Receiver operating characteristic (ROC) curves of single cTBs, clustered cTBs, and cTB-clusters analyzed in different groups. Counts of: **a** single cTBs, **b** clustered cTBs, and **c** cTB-clusters were analyzed in PAS versus non-PAS in all gestational ages (GA = 6–40 weeks). Area under the curves (AUC) with the optimal cTB count cutoff detecting PAS, as well as the sensitivity and specificity of the assays at the optimal cutoffs are listed for each graph. Source data are provided in the Source Data file.

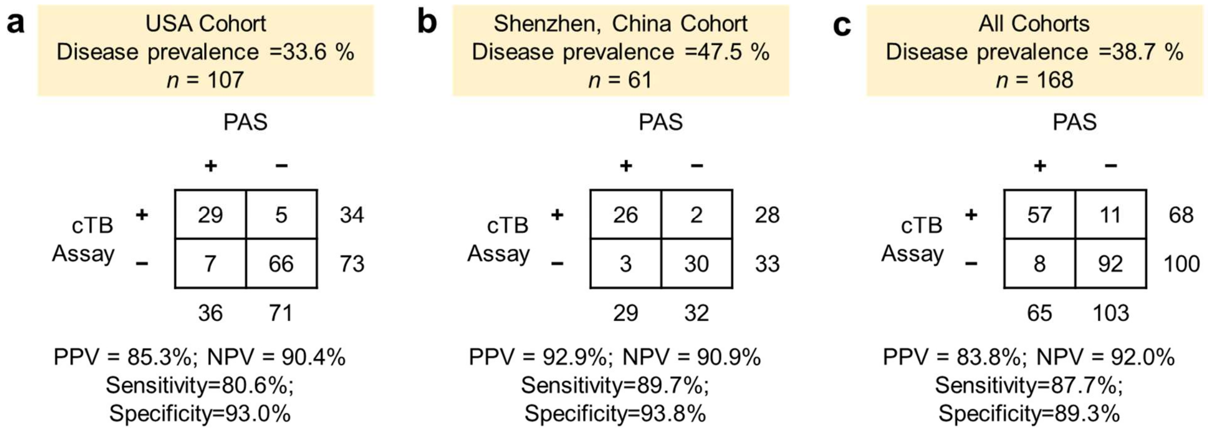

Supplementary Fig. 6. The positive predictive values (PPV) and negative predictive values (NPV) as well as sensitivity and specificity for **a** USA cohort, **b** Shenzhen cohort and **c** all cohorts. The data was entered in a 2×2 table for each analysis.

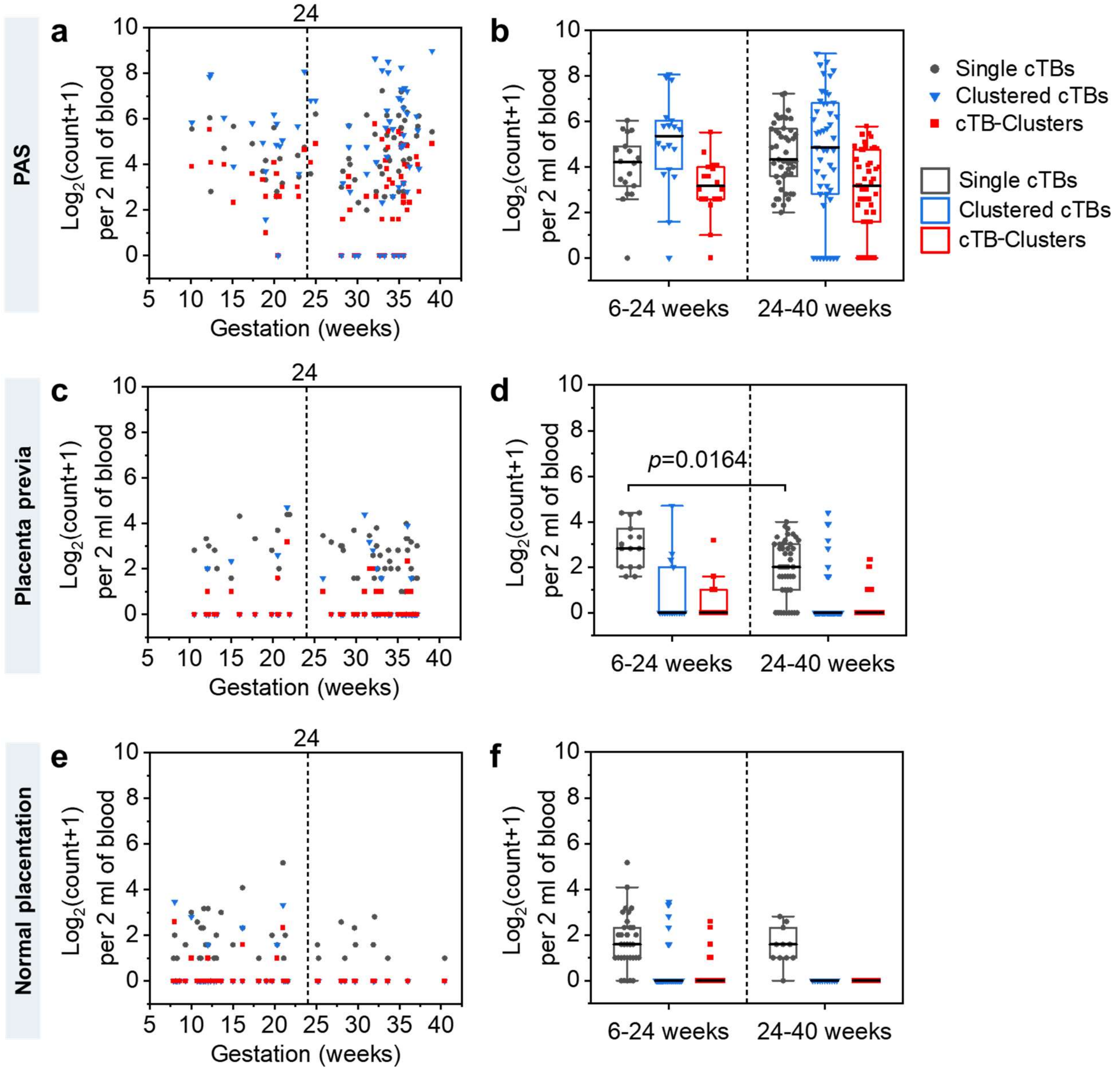

Supplementary Fig. 7. Counts of single cTBs, clustered cTBs, and cTB-clusters based on gestational age for pregnant women with **a**, **b** PAS (n = 65), **c**, **d** placenta previa (n = 59), and **e**, **f** normal placentation (n = 44). Counts were log2-transformed. Box plots show whiskers ranging from minima to maxima, median and 25–75% IQR. Data are expressed as Mean ± SE for **b** PAS < 24 weeks (n = 18): single cTBs (4.0 ± 0.3), clustered cTBs (5.1 ± 0.5), cTB clusters (3.1 ± 0.3); PAS ≥ 24 weeks (n = 47): single cTBs (4.5 ± 0.2), clustered cTBs (4.6 ± 0.4), cTB clusters (3.0 ± 0.3); **d** placenta previa < 24 weeks (n = 15): single cTBs (2.9 ± 0.6), clustered cTBs (0.8 ± 0.4), cTB clusters (0.5 ± 0.2); placenta previa ≥ 24 weeks (n = 44): single cTBs (1.9 ± 0.2), clustered

cTBs (0.6 ± 0.2), cTB clusters (0.3 ± 0.1); and **f** normal placentation < 24 weeks (n = 33): single cTBs (1.7 ± 0.2), clustered cTBs (0.5 ± 0.2), cTB clusters (0.3 ± 0.1); normal placentation ≥ 24 weeks (n = 11): single cTBs (1.5 ± 0.2), clustered cTBs (0.0 ± 0.0), cTB clusters (0.0 ± 0.0). Comparisons of the enumeration data between earlier GA and late GA are evaluated using Mann-Whitney test. All tests are two-sided without adjustments. Source data are provided in the Source Data file.

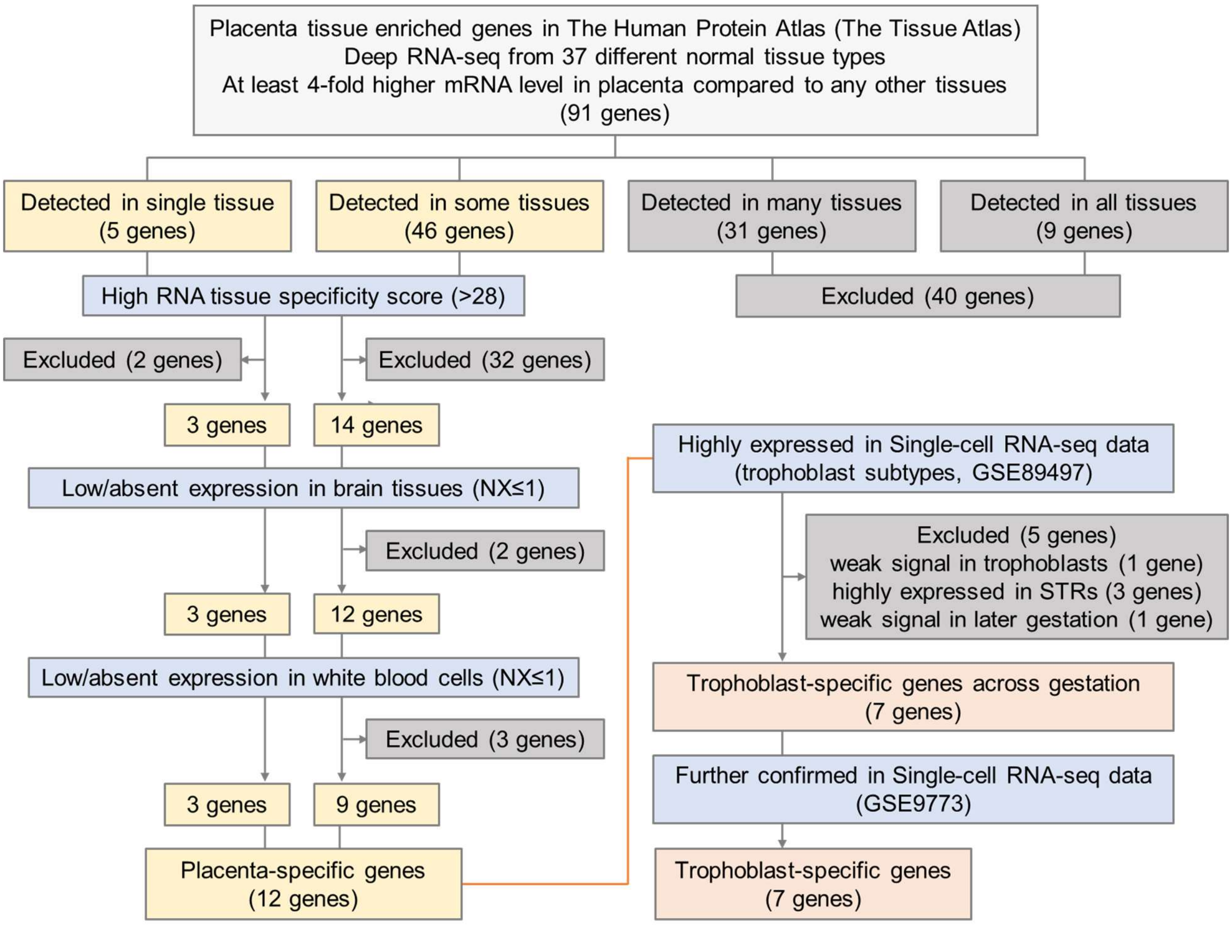

Supplementary Fig. 8. Flowchart for selecting the placenta-specific genes and trophoblast-specific genes. Placenta enriched genes (91 genes) were first identified from The Human Protein Atlas (The Tissue Atlas) database, where the transcript profiling was based on a combination of three transcriptomics datasets (HPA, GTEx, and FANTOM5), corresponding to a total of 483 samples from 37 different human normal tissue types. Placenta-specific genes were subsequently identified among the placenta enriched genes that had high expression in placenta compared to other tissues (including brain and white blood cells) (12 genes). Trophoblast-specific genes were then identified among the placenta-specific genes using single-cell RNA-sequencing datasets. A 7-gene panel that is specific to the trophoblast subpopulation across gestation was identified. Genes that have weak signal in trophoblasts, weak signal in late gestation, or high signal in villous stromal cells (STRs) were excluded. The resulting 7 genes were further confirmed and defined as trophoblast-specific genes.

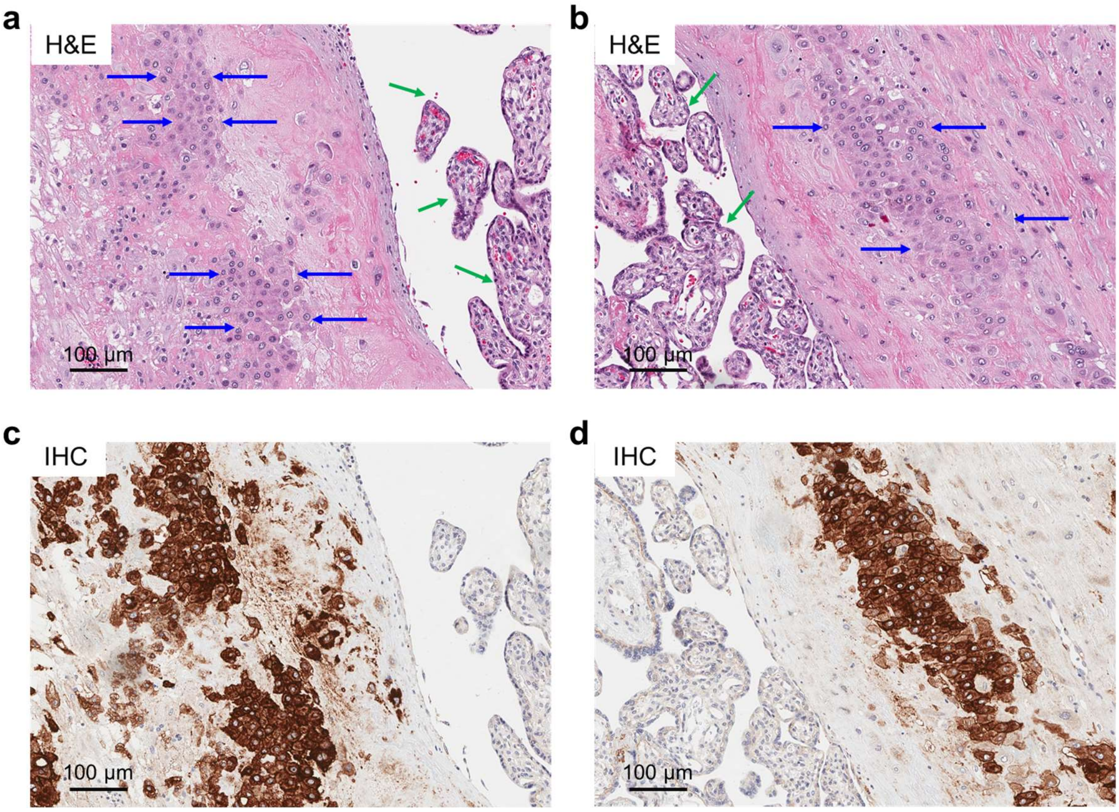

Supplementary Fig. 9. Hematoxylin and Eosin (H&E) staining and immunohistochemistry (IHC) staining of placenta tissues of PAS patients. **a**, **b** Representative images of H&E staining. The blue arrows indicate the extravillous trophoblast cells and the green arrows indicate the chronic villi. **c**, **d** Immunohistochemistry staining of HLA-G in extravillous trophoblast cells (brown color indicates HLA-G positive). HLA-G negative chronic villi tissue serves as the internal negative control. Scale bar, 100 µm. Data are representatives of three independent assays.

Supplementary Table 1. Clinical information for healthy non-pregnant female donors (*n* = 15) .

Characteristics	Healthy non-pregnant female donors
Median maternal age (range)-yo	38 (26–42)
Gravidity-*n* (%)	
0	8 (53.3)
1	3 (20.0)
2	2 (13.3)
≥3	2 (13.3)
Parity-*n* (%)	
0	8 (53.3)
1	4 (26.7)
≥2	3 (20.0)

Supplementary Table 2. Information for the multivariate logistic regression analysis.

Multivariate logistic regression results (PAS versus non-PAS)			
Logistic regression model-stepwise	Odds ratio	95% CI	*p* value
cTB assay-training cohort (Fig. 6a, AUC = 0.947)			
cTB-clusters	1.710	1.264–2.314	< 0.0001
Single cTBs	1.130	1.013–1.260	0.0280
Clustered cTBs	Not included in the model		
cTB assay-all gestational age (Fig. 6d-blue line, AUC = 0.942)			
cTB-clusters	1.760	1.356–2.284	< 0.0001
Single cTBs	1.093	1.014–1.177	0.0195
Clustered cTBs	Not included in the model		
cTB assay+ultrasound-all gestational age (Fig. 6d-orange line, AUC = 0.978)			
Clustered cTBs	1.231	1.114–1.360	< 0.0001
Ultrasound	88.8	18.71–421.4	< 0.0001
Single cTBs, cTB-clusters	Not included in the model		
cTB assay-earlier gestational age (Fig. 6e-blue line, AUC = 0.924)			
cTB-clusters	1.937	1.400–2.679	< 0.0001
Single cTBs, clustered cTBs	Not included in the model		
cTB assay+ultrasound-earlier gestational age (Fig. 6e-orange line, AUC = 0.976)			
cTB-clusters	1.888	1.234–2.887	0.0034
Ultrasound	39.76	3.924–402.8	0.0018
Single cTBs, clustered cTBs	Not included in the model		
cTB assay-late gestational age (Fig. 6f-blue line, AUC = 0.961)			
cTB-clusters	2.090	1.311–3.331	0.0019
Single cTBs	1.282	1.078–1.525	0.0050
Clustered cTBs	Not included in the model		
cTB assay+ultrasound-late gestational age (Fig. 6f-orange line, AUC = 0.979)			
cTB-clusters	3.085	1.493–6.377	0.0024
Ultrasound	145.7	12.08–1758	0.0001
Single cTBs, clustered cTBs	Not included in the model		
cTB assay+clinical factors-all gestational age (AUC = 0.978)			
Clustered cTBs	1.231	1.114–1.360	< 0.0001
Ultrasound	88.8	18.71–421.4	< 0.0001
Single cTBs, cTB-clustereds, Maternal age, BMI, previous CD, Gravidity, Parity, IVF	Not included in the model		

For the stepwise multivariate logistic regression model, variables are entered if $p < 0.05$, variables are removed if $p > 0.1$. CD, cesarean delivery; BMI, body mass index; IVF, in vitro fertilization. CI: confidence intervals.

Supplementary Table 3. Summary of PAS patients (*n* = 65) with/without placenta previa, previous cesarean delivery (CD), and hysterectomy.

Previa	Previous CD	Cases (%)	Hysterectomy	Cases
Yes	Yes	43 (66%)	Yes	32 (49%)
Yes	No	10 (15%)	No	33 (51%)
No	Yes	7 (11%)		
No	No	5 (8%)		

Supplementary Table 4. Detailed information for the 7 trophoblast-specific genes.

Gene	Gene synonym	Gene description	Chromosome	RNA tissue specificity score	RNA tissue-specific NX (placenta)
PSG1	CD66f, PBG1, PSBG1, PSGGA	Pregnancy specific beta-1-glycoprotein 1	19	362	497.6
PSG3		Pregnancy specific beta-1-glycoprotein 3	19	146	495.8
CSH2	CS-2, CSB, hCS-B	Chorionic somatomammot-ropin hormone 2	17	36	246.2
PAPPA2	PAPP-A2, PAPPE, PLAC3	Pappalysin 2	1	35	406.4
CSH1	CSA, CSMT, FLJ75407, hCS-A, PL	Chorionic somatomammot-ropin hormone 1	17	29	450.4
PSG11	MGC22484, PSG13, PSG14	Pregnancy specific beta-1-glycoprotein 11	19	220	151.1
PSG2	CEA, PSBG2, PSG1, PSGGB	Pregnancy specific beta-1-glycoprotein 2	19	102	75.3

Tissue specificity score (TS) defines the fold-change between the expression level in the placenta and the tissue with the second-highest expression level. The mRNA transcript level of each gene in the placenta are shown as normalized expression (NX) values (transcript detectable level describes as NX≥1).

Data are summarized based on the publicly available human placenta transcriptome datasets through the Human Protein Atlas (https://www.proteinatlas.org/humanproteome/tissue/placenta).

Supplementary Table 5. Primers and probes of predesigned Taqman assays (Thermo Fisher Scientific) for each gene.

Gene	Channel color	Assay ID	Catalog NO.
CSH1/2	FAM	Hs04190924_gH	4448892
PSG1	FAM	Hs04235345_s1	4448892
PSG2	FAM	Hs03006946_m1	4448892
PSG3	VIC	Hs02743230_m1	4448489
PSG11	VIC	Hs00601306_m1	4448489
PAPPA2	VIC	Hs00535718_m1	4448489

Supplementary Note 1

Leave-one-out cross validation for the logistic regression model was conducted in R studio (Version 1.4.1103-4). The code for Leave-one-out cross validation for the logistic regression model is shown as following:

The package used is 'caret' https://cran.r-project.org/web/packages/caret/caret.pdf

The functions used :

```
train.control <- trainControl(method = "LOOCV")

model <- train(HCC_OR_NOT ~., data = MY_DATA, method = "bayesglm", trControl = train.control)

x <- predict(model)
```
