# Supplementary material for: Circulating trophoblast cell clusters for early detection of placenta accreta spectrum disorders
Source: Nat Commun. 2021 Aug 3;12:4408. doi: 10.1038/s41467-021-24627-2 (PMC8333096; doi:10.1038/s41467-021-24627-2)
Supplement: Supplementary file 3 — Reporting Summary [file 41467_2021_24627_MOESM3_ESM.pdf]

nature research

Corresponding author(s): Hsian-Rong Tseng

Last updated by author(s): May 21, 2021

Reporting Summary

Nature Research wishes to improve the reproducibility of the work that we publish. This form provides structure for consistency and transparency in reporting. For further information on Nature Research policies, see our Editorial Policies and the Editorial Policy Checklist.

Statistics

For all statistical analyses, confirm that the following items are present in the figure legend, table legend, main text, or Methods section.

n/a	Confirmed	
☐	☒	The exact sample size (*n*) for each experimental group/condition, given as a discrete number and unit of measurement
☐	☒	A statement on whether measurements were taken from distinct samples or whether the same sample was measured repeatedly
☐	☒	The statistical test(s) used AND whether they are one- or two-sided<br>*Only common tests should be described solely by name; describe more complex techniques in the Methods section.*
☐	☒	A description of all covariates tested
☐	☒	A description of any assumptions or corrections, such as tests of normality and adjustment for multiple comparisons
☐	☒	A full description of the statistical parameters including central tendency (e.g. means) or other basic estimates (e.g. regression coefficient) AND variation (e.g. standard deviation) or associated estimates of uncertainty (e.g. confidence intervals)
☐	☒	For null hypothesis testing, the test statistic (e.g. *F*, *t*, *r*) with confidence intervals, effect sizes, degrees of freedom and *P* value noted<br>*Give P values as exact values whenever suitable.*
☒	☐	For Bayesian analysis, information on the choice of priors and Markov chain Monte Carlo settings
☒	☐	For hierarchical and complex designs, identification of the appropriate level for tests and full reporting of outcomes
☐	☒	Estimates of effect sizes (e.g. Cohen's *d*, Pearson's *r*), indicating how they were calculated

Our web collection on statistics for biologists contains articles on many of the points above.

Software and code

Policy information about availability of computer code

Data collection	Fluorescence images and size measurement of cells were collected using the NIS-Elements AR 4.20 software package.<br>Droplet Digital PCR data were collected from QuantaSoft software (Bio-Rad, Hercules, CA)
Data analysis	Multiple comparisons among different groups were evaluated using one-way ANOVA.<br>The statistical tests in this study were performed using the Graphpad Prism 9 (https://www.graphpad.com/) or MedCalc software (version 18.2.1). Leave one out cross validation for the logistic regression model was conducted using R studio.

For manuscripts utilizing custom algorithms or software that are central to the research but not yet described in published literature, software must be made available to editors and reviewers. We strongly encourage code deposition in a community repository (e.g. GitHub). See the Nature Research guidelines for submitting code & software for further information.

Data

Policy information about availability of data

All manuscripts must include a data availability statement. This statement should provide the following information, where applicable:

- Accession codes, unique identifiers, or web links for publicly available datasets
- A list of figures that have associated raw data
- A description of any restrictions on data availability

All data that support the findings and conclusions of this paper are present in the paper and/or the Supplementary Information. Additional data related to this paper are available on request from the authors.

Field-specific reporting

Please select the one below that is the best fit for your research. If you are not sure, read the appropriate sections before making your selection.

- [x] Life sciences
- [ ] Behavioural & social sciences
- [ ] Ecological, evolutionary & environmental sciences

For a reference copy of the document with all sections, see nature.com/documents/nr-reporting-summary-flat.pdf

Life sciences study design

All studies must disclose on these points even when the disclosure is negative.

Sample size	The study size was prespecified.
Data exclusions	Women were excluded if they are diagnosed with known or suspected fetal genetic/congenital abnormalities or blood draw was not possible.
Replication	All attempts at replication in the cell line study were successful.
Randomization	N/A
Blinding	All laboratory samples were assayed by investigators blinded to the clinical status of the subjects.

Reporting for specific materials, systems and methods

We require information from authors about some types of materials, experimental systems and methods used in many studies. Here, indicate whether each material, system or method listed is relevant to your study. If you are not sure if a list item applies to your research, read the appropriate section before selecting a response.

Materials & experimental systems

n/a	Involved in the study	
[ ]	[x]	Antibodies
[ ]	[x]	Eukaryotic cell lines
[x]	[ ]	Palaeontology and archaeology
[x]	[ ]	Animals and other organisms
[ ]	[x]	Human research participants
[x]	[ ]	Clinical data
[x]	[ ]	Dual use research of concern

Methods

n/a	Involved in the study	
[x]	[ ]	ChIP-seq
[x]	[ ]	Flow cytometry
[x]	[ ]	MRI-based neuroimaging

Antibodies

Antibodies used

Biotinylated goat anti-EpCAM (R&D Systems, Catalog #BAF960)
Rabbit anti-CK7 (Abcam, Catalog #ab53123)
Mouse HLA-G (Abcam, Catalog #ab52455)
Rat anti-CD45 (ThermoScientific, Catalog #MA5-17687)
Anti-rabbit IgG (H+L), Alexa 488 conjugated (Fisher Scientific, Catalog #A21206)
Anti-mouse IgG (H+L), Alexa 555 conjugated (Fisher Scientific, Catalog #A31570)
Anti-Rat IgG (H+L), Alexa 647 conjugated (Abcam, Catalog #ab150155)

Validation

Only commercial and validated antibodies have been used. The validation of each primary antibody for the species and application can be found on the following manufacturer websites:
Biotinylated goat anti-EpCAM, https://www.rndsystems.com/products/human-epcam-trop-1-biotinylated-antibody_baf960
Rabbit anti-CK7, https://www.abcam.com/cytokeratin-7-antibody-ab53123.html
Mouse HLA-G, https://www.abcam.com/hla-g-antibody-4h84-ab52455.html
Rat anti-CD45, https://www.thermofisher.com/antibody/product/CD45-Antibody-clone-YAML501-4-Monoclonal/MA5-17687
Anti-rabbit IgG (H+L), Alexa 488 conjugated, https://www.thermofisher.com/antibody/product/Donkey-anti-Rabbit-IgG-H-L-Highly-Cross-Adsorbed-Secondary-Antibody-Polyclonal/A-21206
Anti-mouse IgG (H+L), Alexa 555 conjugated, https://www.thermofisher.com/antibody/product/Donkey-anti-Mouse-IgG-H-L-Highly-Cross-Adsorbed-Secondary-Antibody-Polyclonal/A-31570
Anti-Rat IgG (H+L), Alexa 647 conjugated, https://www.abcam.com/donkey-rat-igg-hl-alexa-fluor-647-preadsorbed-ab150155.html

Eukaryotic cell lines

Policy information about cell lines

Cell line source(s)	JEG-3 cell line was purchased from ATCC.
Authentication	Authentication was performed by analysis of morphology.
Mycoplasma contamination	JEG-3 cell line was tested negative for mycoplasma contamination.
Commonly misidentified lines (See ICLAC register)	No commonly misidentified lines were used in this study.

Human research participants

Policy information about studies involving human research participants

Population characteristics	We collected blood samples from 168 individuals in four pregnant cohorts and one non-pregnant cohort, (i) PAS cohort: prenatally suspected and subsequently pathologically confirmed PAS patients (n = 65, mean age = 36 years old (yo)); (ii) placenta previa cohort: clinically diagnosed placenta previa patients (n = 59, mean age = 35 yo); (iii) normal placentation cohort: pregnant women with clinically confirmed normal placentation (n = 44, mean age = 37 yo); and (iv) healthy non-pregnant female donors (n = 15, mean age = 29 yo). Detailed chracteristics could be found in Table 1 and Supporting Information (Supplementary Table 1).
Recruitment	Pregnant women aged from 18 years old to 45 years old with singleton intrauterine pregnancies, and gestational ages between 6 and 40 weeks were eligible for inclusion. Samples were collected from December 2017–January 2021) during prenatal care visits. Women were excluded if they are diagnosed with known or suspected fetal genetic/congenital abnormalities or blood draw was not possible. Pregnant women were classified as normal placentation, placenta previa (without placenta accreta), and PAS. PAS or placenta previa was defined and diagnosed according to current American College of Obstetricians and Gynecologists (ACOG) and Society for Maternal-Fetal Medicine (SMFM) guidelines as well as FIGO consensus guidelines.
Ethics oversight	This observational cohort study protocol was approved by the Institutional Review Boards (IRB) of University of California, Los Angeles (UCLA) (UCLA IRB#13-001264), Cedars-Sinai Medical Center (CSMC) (CSMC IRB #Pro00006806 and Pro00008600) and Shenzhen People's Hospital (LL-KY-2019608).

Note that full information on the approval of the study protocol must also be provided in the manuscript.
